# Burden of hospital admissions and resulting patient interhospital transports during the 2020/2021 SARS-CoV-2 pandemic in Saxony, Germany

**DOI:** 10.1038/s41598-023-35406-y

**Published:** 2023-05-24

**Authors:** Katrin Bender, Felix Waßer, Yacin Keller, Ulrich Pankotsch, Hanns-Christoph Held, Robin R. Weidemann, Christian Kleber, Christoph Höser, Sebastian N. Stehr

**Affiliations:** 1 Department of Anesthesiology and Critical Care Medicine, University Hospital Leipzig, Leipzig, Germany; 2 GeoHealth Centre, Institute for Hygiene and Public Health, University Hospital Bonn, Bonn, Germany; 3 City of Dresden Fire Department, Integrated Regional Control Centre, Dresden, Germany; 4 Department of Visceral, Thoracic and Vascular Surgery, University Hospital Carl Gustav Carus, Dresden, Germany; 5 University Hospital Carl Gustav Carus Dresden, Dresden, Germany; 6 Department of Orthopaedic and Trauma Surgery, University Hospital Carl Gustav Carus, Dresden, Germany; 7 Department of Orthopaedic and Trauma Surgery, University Hospital Leipzig, Leipzig, Germany

**Keywords:** Health services, Public health

## Abstract

Secondary transports of patients from one hospital to another are indicated for medical reasons or to address local constraints in capacity. In particular, interhospital transports of critically ill infectious patients present a logistical challenge and can be key in the effective management of pandemic situations. The state of Saxony in Germany has two characteristics that allow for an extensive evaluation of secondary transports in the pandemic year 2020/2021. First, all secondary transports are centrally coordinated by a single institution. Second, Saxony had the highest SARS-CoV-2 infection rates and the highest COVID-19 associated mortality in Germany. This study evaluates secondary interhospital transports from March 2019 to February 2021 in Saxony with a detailed analysis of transport behaviour during the pandemic phase March 2020 to February 2021. Our analysis includes secondary transports of SARS-CoV-2 patients and compares them to secondary transports of non-infectious patients. In addition, our data show differences in demographics, SARS-CoV-2- incidences, ICU occupancy of COVID-19 patients, and COVID-19 associated mortality in all three regional health clusters in Saxony. In total, 12,282 secondary transports were analysed between March 1st, 2020 and February 28th, 2021, of which 632 were associated with SARS-CoV-2 (5.1%) The total number of secondary transports changed slightly during the study period March 2020 to February 2021. Transport capacities for non-infectious patients were reduced due to in-hospital and out-of-hospital measures and could be used for transport of SARS-CoV-2 patients. Infectious transfers lasted longer despite shorter distance, occurred more frequently on weekends and transported patients were older. Primary transport vehicles were emergency ambulances, transport ambulances and intensive care transport vehicles. Data analysis based on hospital structures showed that secondary transports in correlation to weekly case numbers depend on the hospital type. Maximum care hospitals and specialized hospitals show a maximum of infectious transports approximately 4 weeks after the highest incidences. In contrast, standard care hospitals transfer their patients at the time of highest SARS-CoV-2 case numbers. Two incidence peaks were accompanied by two peaks of increased secondary transport. Our findings show that interhospital transfers of SARS-CoV-2 and non-SARS-CoV-2 patients differ and that different hospital care levels initiated secondary transports at different times during the pandemic.

## Introduction

The first positive SARS-CoV-2 case in Germany was diagnosed at the end of January 2020 in Bavaria (German patient zero)^1^. From this time point on, the virus spread rapidly across Germany and affected the federal states differently. Due to strict lockdown measures, Saxony was most probably able to effectively confine the virus and thus had an incidence below the national average^2^.

In March 2020 the responsible government institution (Saxon Ministry of Social Affairs), divided Saxony into three COVID-19 coordination clusters—Chemnitz, Dresden and Leipzig^3^. One hospital (University Hospital Dresden, University Hospital Leipzig and the Hospital Chemnitz) in each cluster served as a central coordinator for SARS-CoV-2 patients and as a contact for outpatient and inpatient stakeholders in the healthcare system as well as for public health authorities^4^. Up to that point, direct data exchange and communication between hospitals were not standard. In the German health care system, hospitals act as individual players and operate within their own structures and hierarchies. As of March 2020 hospitals in Saxony were expected to function as a single entity, sharing responsibility for SARS-CoV-2/COVID-19 patients in a regional cluster. By means of centralised management, each cluster was primarily responsible for the care of infected patients in the respective region and was expected to counteract local and regional overloads. If COVID-19 capacities in a cluster were exhausted, patients were transferred to the other two clusters. Relocation within defined clusters has also been successfully applied in other federal states^5^. Across Germany, the so-called “cloverleaf concept” was developed early in the COVID-19 pandemic as a measure to buffer regional overload. The 16 federal states were grouped into 5 regions (north, west, south, east and southwest) in the cloverleaf coordination. Transport within the framework took place over longer distances (up to 1000 km) or a longer period of time (approx. 6 to 12 h transport duration) and with changing means of transport. Aim was a timely transfer with the patient's well-being and clinical picture in mind^6,7^. In autumn 2020, incidences all over Germany started to surge and particularly in Saxony, there was a dramatic increase in the number of infections. By Christmas, the state had the highest incidences in Germany^8^. Local health care providers in high-incidence areas reached their capacity limits for SARS-CoV-2/ COVID-19 patients and could not provide adequate care.

Capacity constraints can be addressed in various ways. Adding staff and beds to normal or intensive care units is usually possible only—if at all—for large hospitals such as maximum care hospitals. Even then, normal hospital operations have to be reduced^9^. The second option is to consider transfers of patients to other hospitals (both SARS-CoV-2 positive and negative).

Secondary transports of SARS-CoV-2 patients may be necessary for several reasons: (1) separation and isolation of infected patients; (2) insufficient hospital bed capacity to handle the flood of infected patients (quantitative secondary transport) or (3) escalation of care due to medical limits and the need to access specialist treatment not available in the referring hospital (e.g. ECMO therapy, qualitative secondary transport). COVID-19 patients with a severe course of disease and impending multi-organ failure were often transferred to higher level hospitals to increase the chance of survival.

The evaluation of SARS-CoV-2 associated transport data on this scale is unique to date. Our motivation for evaluating secondary transport data was multifaceted. A direct comparison with non-infectious secondary transports may provide patient-specific differences. The authors work at a maximum care hospital (ARDS- centre with the option of ECMO- therapy) and there was evidence that COVID-19 patients who received secondary transfers tended to be older. This was perceived data with no scientific data basis. In addition, the mode of transport may provide clues to the severity of illness. Again, there were only internal case reports at the author’s hospital that COVID-19 patients were significantly sicker. By analysing the departure and arrival hospitals, we hoped to obtain information on which hospital care levels were more burdened with infectious and non- infectious secondary transports, respectively.

Our aim of this paper is the evaluation of the impact of the SARS-CoV-2 outbreak on secondary transports of patients.

## Methods

### Study design

We performed a retrospective analysis of secondary transports of SARS-CoV-2 positive and negative patients from March 1st, 2019 until February 28th, 2021 in the federal state of Saxony, Germany. This analysis is covered by the ethical approval 242/21-ek of the ethical committee of the Medical Faculty of the University of Leipzig (Ethikkommission der Medizinischen Fakultät der Universität Leipzig, Germany). The Ethics Committee of University of Leipzig has waived the requirement of informed consent. All methods were carried out in accordance with relevant guidelines and regulations.

### Ambulance transport data

Secondary transport data of patients with (detected or suspected) SARS-CoV-2 infections and non- infectious patients were provided by the Integrated Rescue Control Centre (IRCC) in Dresden. The IRCC is responsible for the coordination of all secondary transports in Saxony.

SARS-CoV-2 associated transports require intensive cleaning and disinfection. This applies to transports of patients which are probably infected but not yet confirmed to be negative, too. For this reason the term “COVID-19 transport/SARS-CoV-2 transport,” as used in this paper, applies to proven SARS-CoV-2 infected patients as well as to patients probably infected while their status at time of transport was not yet confirmed.

The original dataset contained data from the year before the pandemic as reference period (March 1st, 2019 to February 28th, 2020) and the pandemic period (March 1st, 2020 to February 28th, 2021). The initial dataset (containing transfers to other states and abroad) consisted of 37,815 secondary transports from March 1st, 2019 to February 28th, 2021. Sporadically, patients were transferred to other federal states and abroad. To define a localised region, we focused on secondary transports with start and destination location within Saxony only. Additionally, we included only those transfers where start and destination are different hospitals. In case a hospital or hospital-group has several locations these transfers are excluded as well. Data clearing identified 185 transports inconsistently represented in the datasets which have been excluded as well. The finalised dataset included 25,175 secondary transports; a total of 12,893 secondary transports in the reference period March 1st, 2019 until February 28th, 2020 and 12,282 secondary transports from March 1st, 2020 until February 28th, 2021. Out of these, 632 secondary transports have been marked as SARS-CoV-2 associated transports, whereby only 2 out of 632 transports were in the reference period.

The dataset includes information about name and location of hospital (start and destination), transfer-specific items like id, time, date, type of vehicle, vehicle’s providing organisation and patient’s age and gender.

This information was enhanced with start- and destination-specific information about the hospitals’ bed capacities, level of care and ownership of the hospital^21^.

The road-distances between all possible locations have been calculated using ArcGIS Pro^Ⓡ^ 3.1. The time spent for each transfer has been taken from the original dataset of transfers and includes the time for handling patients, handover protocols as well as cleaning and disinfection.

### Statistical analysis

The statistical analysis compares secondary transports of infectious and non-infectious patients. Datasets on secondary transports have been transferred into a relational database (MariaDB^Ⓡ^). The graphical analysis compares temporal distribution of incidences and secondary transports and inspects the attributes of those transports.

### Chi-square test

To perform the test, a multi-field table is created containing the absolute frequencies for each combination of the variables under investigation. The null hypothesis is then tested: both variables examined are independent. The test is performed at the 5% significance level and is significant if the *p* value is < 0.05. The independence between SARS-CoV-2 secondary transport (yes/no) and the age distribution, the distribution of the transport type and the distribution on the days of the week is examined using this test.

### Shapiro–wilk test

The test is used to decide whether to use the t-test (if normally distributed) or the Wilcoxon rank sum test (not normally distributed) afterwards. The null hypothesis is: The variable tested can be considered normally distributed. If the test is significant, then a normal distribution cannot be assumed. The test is performed at the 5% significance level and is thus significant if the *p* value is < 0.05.

### Wilcoxon rank sum test (Mann–Whitney U test)

It is a non-parametric test and is used when no normal distribution of the data can be assumed. The test checks whether two variables differ significantly in their position. If the test is significant (here at the 5% level) (*p* value < 0.05), then both variables have a different position. In this manuscript, the test is used to check for a significant difference between the two groups SARS-Cov-2- transfer and NON-SARS-CoV-2 transfer for the variable “age” and “duration”.

### Saxony

Saxony is one of 16 federal states, located in the east of Germany. It is the 10th largest state with an area of 18,449 km^2^ and a population of just over 4 million^10^. Saxony has three territorial units at the NUTS-2 hierarchical level; Chemnitz, Dresden and Leipzig. NUTS-2 Chemnitz and NUTS-2 Dresden each have 5 NUTS-3 levels (counties) and NUTS-2 Leipzig has 3 NUTS-3 levels^11^. Due to different territorial size, NUTS-2 areas have different numbers of acute hospital locations: Chemnitz with 29 hospitals, Dresden with 31 and Leipzig with 17, respectively^12^. NUTS-2 levels correspond to the above-mentioned clusters established by the Saxon Ministry of Social Affairs at the beginning of the first corona wave. For simplicity, the NUTS-2 levels are referred to as clusters in this manuscript.

### Demographic data saxony

The population as well as the average age of the population for each cluster were extracted from the official website of the federal state of Saxony^13^.

The number of hospitals and the number of hospital beds per 100,000 inhabitants were also extracted and calculated from the official website of the state of Saxony, respectively^12^.

The number of intensive care beds [betten_belegt_nur_erwachsene UND betten_frei_nur_Erwachsene] were extracted from a central German database (DIVI)^14^. Free and occupied beds (= total number of ICU beds) for each cluster were determined on a daily basis. The daily values were used to calculate the mean value of hospital beds for the study period from March 1st, 2020 to February 28th, 2021.

### Infection rates and SARS-CoV-2 incidences

The Robert Koch Institute (RKI) is the central institution of the Federal German Government in the field of surveillance and prevention of infectious diseases^15^. The RKI receives daily positive SARS-CoV-2 counts from public health departments. From these numbers, the incidences are calculated for each federal state and each county. The first SARS-CoV-2 case in Saxony was taken from daily reports on infection rates provided by the RKI^16^. SARS-CoV-2 incidences for state comparisons were either retrieved from the RKI website^17^ or obtained from “SurvStat@RKI 2.0” according to own criteria (registration week, cluster, counties, year).

### Mortality data

Mortality data for the period March 1st, 2020 to February 28th, 2021 were requested from the State Statistical Office in Saxony. Total mortality and COVID-19 associated mortality were considered. Mortality due to COVID-19 was filtered out using the ICD-10 classification U07.1, U07.2 (ICD-10: 10th version of the International Statistical Classification of Diseases and Related Health Problems). U07.1: COVID-19, virus detected. U07.2: COVID-19, Virus not detected. COVID-19 associated mortality was calculated as follows: the number of COVID-19 associated deaths per calendar week in each county was summed for each cluster and related to the population of the cluster. COVID-19 associated mortality for state comparison was retrieved from the RKI website^18^.

### Intensive care admission

Data on occupancy rates of COVID-19 patients in intensive care units in Saxony were retrieved from the intensive care register of the “German Interdisciplinary Association for Intensive Care and Emergency Medicine” (DIVI)^14^ and the RKI Available are daily updated data on the occupancy rates of COVID-19 patients in each county from each federal state.

### Hospital classification in Germany

Hospitals are categorised into different levels of care for in-patients. A comparable classification would be the American classification “Level I to V” for trauma centres^19^. A “maximum care hospital” (“Maximalversorger”) is capable of providing total care for every aspect of injury and has full coverage of all operating and non-operating departments. An equivalent would be a Level I Trauma Centre. A “Regelversorger” provides standard care comparable to a Level III hospital. A “Schwerpunktversorger” is an advanced care hospital offering medical expertise that lies between maximum care and standard care hospitals. A „Fachkrankenhaus” is a specialised hospital offering various steps of rehabilitation for all patients of all ages after the acute convalescence.

### Classification of ambulances in Germany

Germany distinguishes different types of ambulances in the medical transport system. Advanced medical equipment as well as extended medical knowledge of crew members distinguish transport vehicles. “Transport ambulances” are often abbreviated KTW (*K*ranken*T*ransport*W*agen) and usually transport patients that do not need acute intensive care. An “emergency ambulance “(rescue transport vehicle or “*R*e*T*tungs*W*agen”, RTW), has additional medical equipment and a wider range of emergency medication compared to transport ambulances and can transport acutely sick patients. If a patient's condition requires more intensive care equipment, the transport vehicle is often referred to as intensive care transport vehicle (ITW) or critical care transport vehicle^20^. In addition, helicopters (*R*e*T*tungs*H*ubschrauber, RTH) are used both for rescue and for medical transport and carry medical equipment equivalent to an emergency ambulance.

## Results

### Demographic data saxony

**Figure 1:**
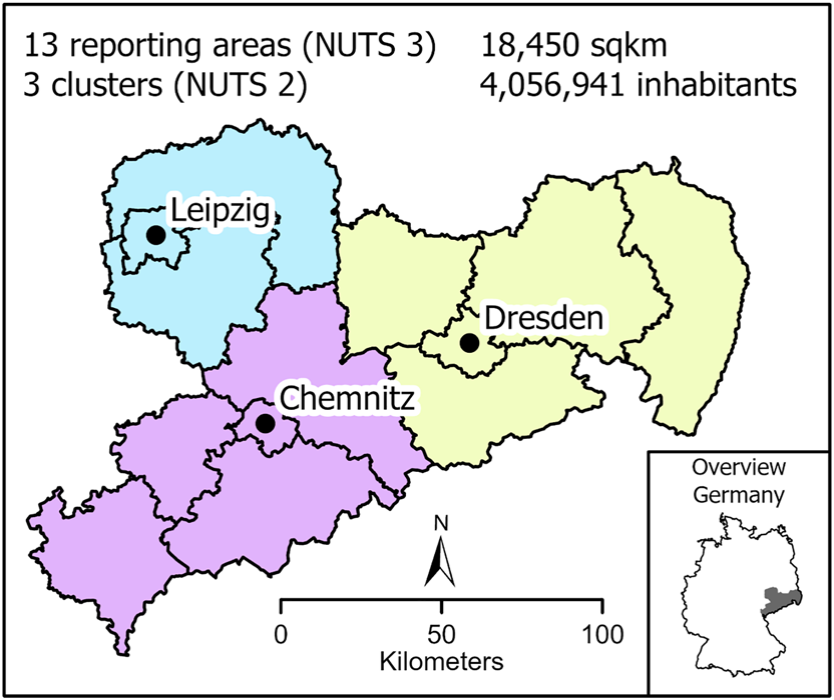
Geographical overview of Saxony within Germany (small map) and the three clusters Chemnitz (lilac), Dresden (yellow) and Leipzig (light blue) with the corresponding cities (black dots).

At the beginning of the coronal pandemic, Saxony was divided into 3 clusters according to the NUTS2 classification (Fig. 1).

The composition of the population in each cluster varies widely. First, the proportion of the population living in the three largest cities Chemnitz, Dresden and Leipzig differs (Table 1). More than half of the population in the Leipzig cluster lives in the city of Leipzig (57%). In contrast, only 17% of the population in the Chemnitz cluster lives in the city of Chemnitz, leaving 83% in rural areas. In the Dresden cluster, 35% of the people live in Dresden and 65% outside the city. Second, the age distribution varies among the clusters. While the average age in the Leipzig cluster is 44.8 years, people are on average 2 years older in the Dresden cluster and 4 years older in the Chemnitz cluster. The share of people > 65 years is highest in the Chemnitz cluster with 29.7%, followed by Dresden with 26.3% and Leipzig with 23.1%.

**Table 1:** General demographic data for the three clusters Chemnitz, Dresden and Leipzig.

Cluster	Population (in million)	% Population in large cities	Average age	% population > 65 y	Hospitals	Hospital beds/100,000 inhabitants	ICU beds/100,000 inhabitants
*Chemnitz*	1.413.730	17.3	48.7	29.7	29	670	35
*Dresden*	1.589.888	35	46.7	26.3	31	615	39
*Leipzig*	1.053.323	57	44.8	23.1	17	620	45
*Sachsen*	4.056.941		46.9	26.7	77		

Health care in the Dresden cluster is provided by 31 hospitals, followed by the Chemnitz cluster with 29 and Leipzig with 17 hospitals. The availability of hospital beds and especially intensive care beds played a major role in 2020/21. The Chemnitz cluster has the highest number of hospital beds with 670 beds and the lowest number of intensive care beds (35 ICU beds) (both per 100,000 inhabitants). The Leipzig cluster has 620 hospital beds and 45 intensive care beds; the Dresden cluster has 615 hospital beds and 39 intensive care beds (Table 1)^21^.

### Development of regional SARS-CoV-2 infections in Saxony

#### Infection/incidence data: reported cases

**Figure 2:**
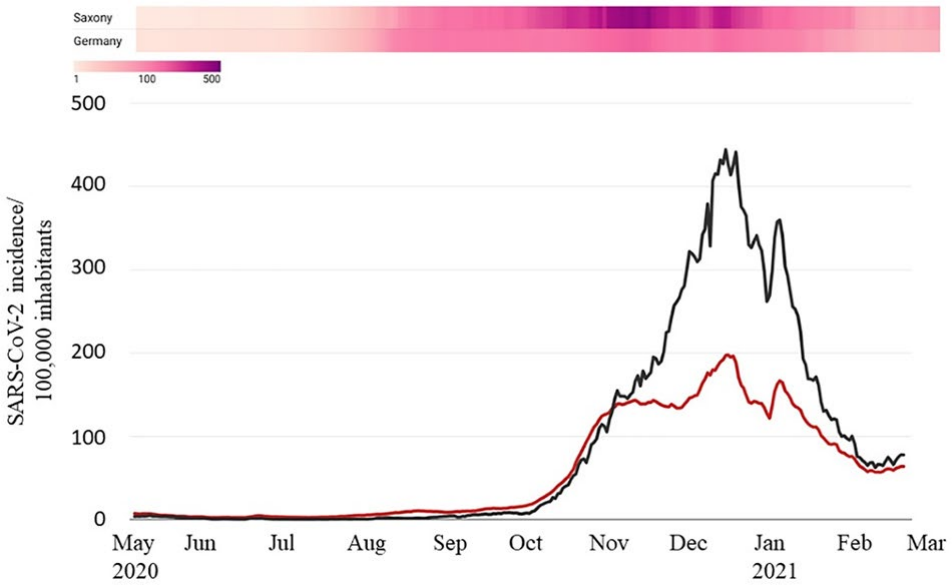
7-days incidences of SARS-CoV-2 infections in Germany (red) and Saxony (black) from May 2020 to February 2021 and the corresponding heatmap (top).

The first SARS-CoV-2 case in Saxony was reported in the district of *Saxon Switzerland- Eastern Ore Mountains* in early March, marking the beginning of the first wave. Compared to the national average, the number of infections in Saxony during the first wave was constantly below 5 while incidences in other federal states such as Bremen were around 25. With the onset of the second wave in fall 2020, the incidence in Saxony changed dramatically compared to the overall incidence in Germany. The number of infections increased steadily from October onward, and by the end of November, Saxony had the highest incidences nationwide (beginning November 27, 2020, until January 12, 2021). Reported infections peaked around Christmas with a maximum incidence of 444 in Saxony, while the nationwide incidence was 197 (data on December 27, 2020) (as shown in Fig. 2).

As illustrated in Table 2 (top), the three clusters differed in their peak incidences. The Dresden cluster had the highest incidence of 550/100,000 inhabitants (calendar week 51 in 2020), the Chemnitz cluster of 518/100,000 inhabitants and the Leipzig cluster of 363/100,000 inhabitants. With the onset of the second wave and the increase in SARS-CoV-2 incidences, the occupancy rates of COVID-19 patients in intensive care units also began to increase in Saxony (Table 2 (bottom)). At the peak of the second wave in December 2020/ January 2021, 19 COVID-19 patients (per 100,000 inhabitants) were treated in the Dresden cluster, 14 in the Chemnitz and 10 in the Leipzig cluster (both per 100,000 inhabitants). The average incidence of COVID-19 patients treated in intensive care units in Germany during the same period was approximately 7/100,000 inhabitants. With an incidence average of 15 COVID-19 patients per 100,000 inhabitants, Saxony had the highest occupancy rate in intensive care units nationwide.

**Table 2:** 7-days incidence and COVID-19 patients in ICU (both per 100,000 inhabitants) in Saxony and the three clusters Chemnitz, Dresden and Leipzig between the end of August 2020 (KW 36) until the end of February 2021 (KW 8).

7 days incidence/100,000 inhabitants	KW 36	KW 43	KW 47	KW 49	KW 51	KW 53	KW 1	KW 3	KW 4	KW 8
Saxony	4.8	86.4	222.9	344.6	493.0	341.6	374.4	176.1	128.2	88.3
Chemnitz	2.9	103.5	254.5	372.6	518.5	364.7	336.5	155.1	114.9	101.2
Dresden	3.8	94.1	255.4	411.0	550.2	369.9	441.6	187.6	136.8	77.1
Leipzig	9.4	48.3	123.6	196.2	363.1	261.7	323.7	189.0	134.5	86.7

COVID-19 patients in ICU/ 100,000 inhabitants	KW 36	KW 43	KW 47	KW 49	KW 51	KW 53	KW 1	KW 3	KW 4	KW 8
Saxony	0.1	1.3	6.7	9.3	13.3	13.9	13.3	11.8	10.2	5.6
Chemnitz	0.0	2.1	6.4	9.1	12.4	13.4	14.4	12.0	9.7	4.9
Dresden	0.4	1.1	9.8	12.6	18.5	17.9	15.1	13.1	11.2	6.4
Leipzig	0.0	0.5	2.5	4.7	6.6	8.6	9.1	9.4	9.5	5.2

In parallel with rising infection numbers in fall 2020, COVID-19 associated deaths also increased (Table 3). Around Christmas, the Leipzig cluster reported the highest COVID-19 associated mortality of 14/100,000 inhabitants. The Chemnitz and Dresden clusters showed an even higher mortality of nearly 27 COVID-19 associated deaths/100,000 inhabitants. In addition, a direct comparison with the average overall mortality in Saxony in 2016–2019 shows a nearly 2.5-fold increase in mortality during the pandemic period.

**Table 3:** Overall mortality and COVID-19 associated death (both per 100,000 inhabitants) in Saxony (2016–2019 and 2020–2021) and the three clusters Chemnitz, Dresden and Leipzig between the end of August 2020 (KW 36) until the end of February 2021 (KW 8).

Overall mortality/100,000 inhabitants	KW 36	KW 43	KW 47	KW 49	KW 51	KW 53	KW 1	KW 3	KW 4	KW 8
Saxony 2016–2019	23.3	24.5	25.2	26.4	27.6	27.6	27.8	27.7	28.9	30.8
Saxony 2020–2021	24.8	28.0	37.3	44.8	58.8	58.3	51.1	42.1	38.0	28.0
Chemnitz	30.5	32.2	43.4	52.8	70.3	68.9	58.9	48.0	44.0	30.6
Dresden	22.0	26.3	38.2	46.8	63.6	59.4	50.0	39.6	37.4	27.0
Leipzig	21.5	24.8	27.9	31.0	36.1	42.2	42.2	38.2	30.7	25.9

COVID-19 associated death/100,000 inhabitants	KW 36	KW 43	KW 47	KW 49	KW 51	KW 53	KW 1	KW 3	KW 4	KW 8
Saxony	0.0	1.0	7.0	11.8	22.3	21.1	17.9	12.1	10.5	3.7
Chemnitz	0.0	2.3	8.2	14.2	26.9	24.0	20.8	12.9	11.1	3.8
Dresden	0.0	0.6	9.4	14.0	27.3	23.6	18.2	13.1	12.0	4.0
Leipzig	0.0	0.0	1.7	5.2	8.6	13.6	13.5	9.5	7.5	2.9

### Ambulance transfers

**Figure 3:**
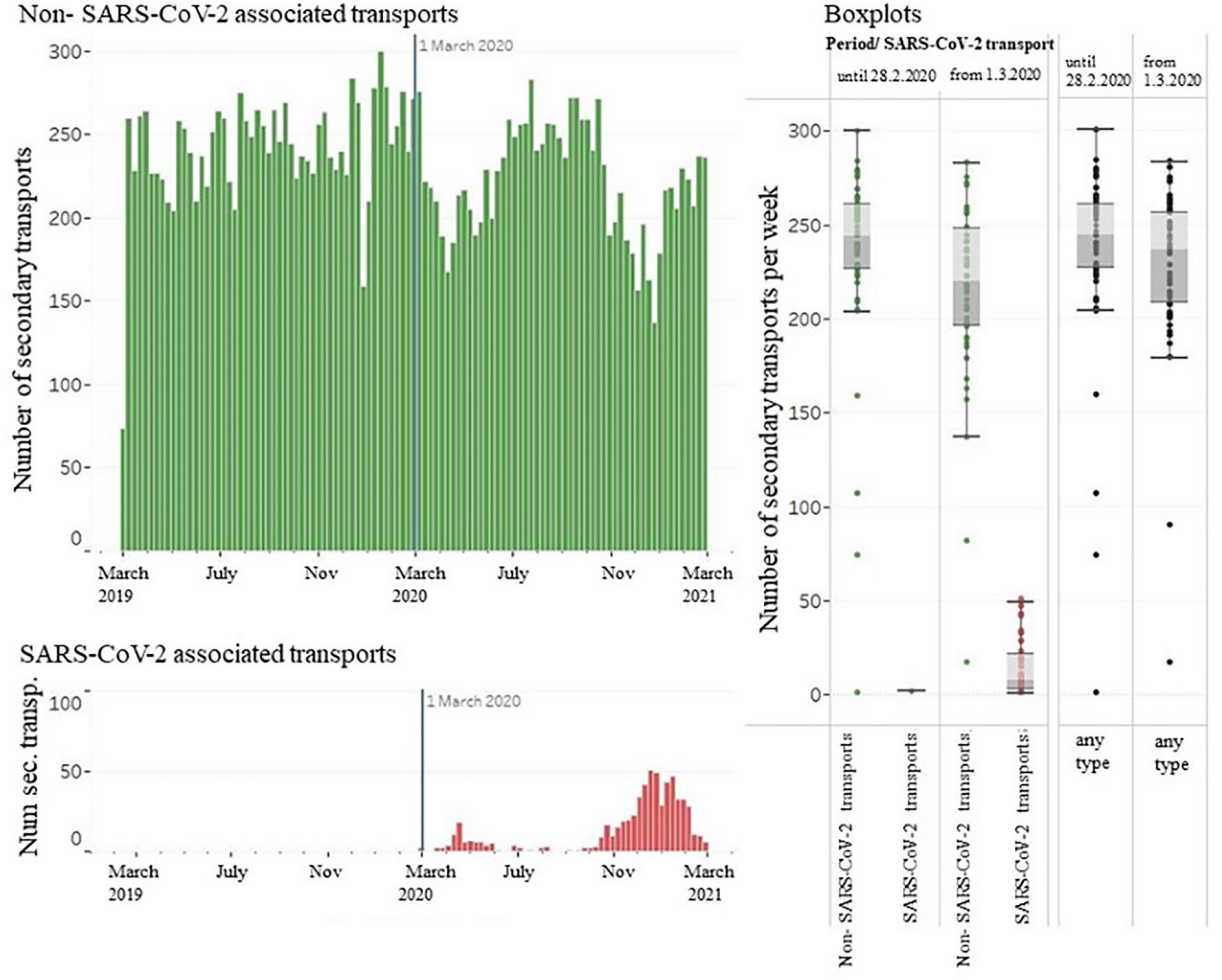
Total number of weekly secondary transports of SARS-CoV-2 positive patients (red columns) and SARS-CoV-2 negative patients (green columns) from the reference period March 1st, 2019 until February 28th, 2020 and the pandemic phase March 1st, 2020 until February 28th, 2021. The vertical blue line marks the end of our reference period.

Figure 3 illustrates SARS-CoV-2 and non-SARS-CoV-2 associated transports over time. Secondary transports of infected patients correlated with the first and second corona wave. The number of all secondary transports as well as secondary transports of non-infectious patients were slightly reduced in the pandemic period March 1st, 2020 until February 28th, 2021 in Saxony.

**Figure 4:**
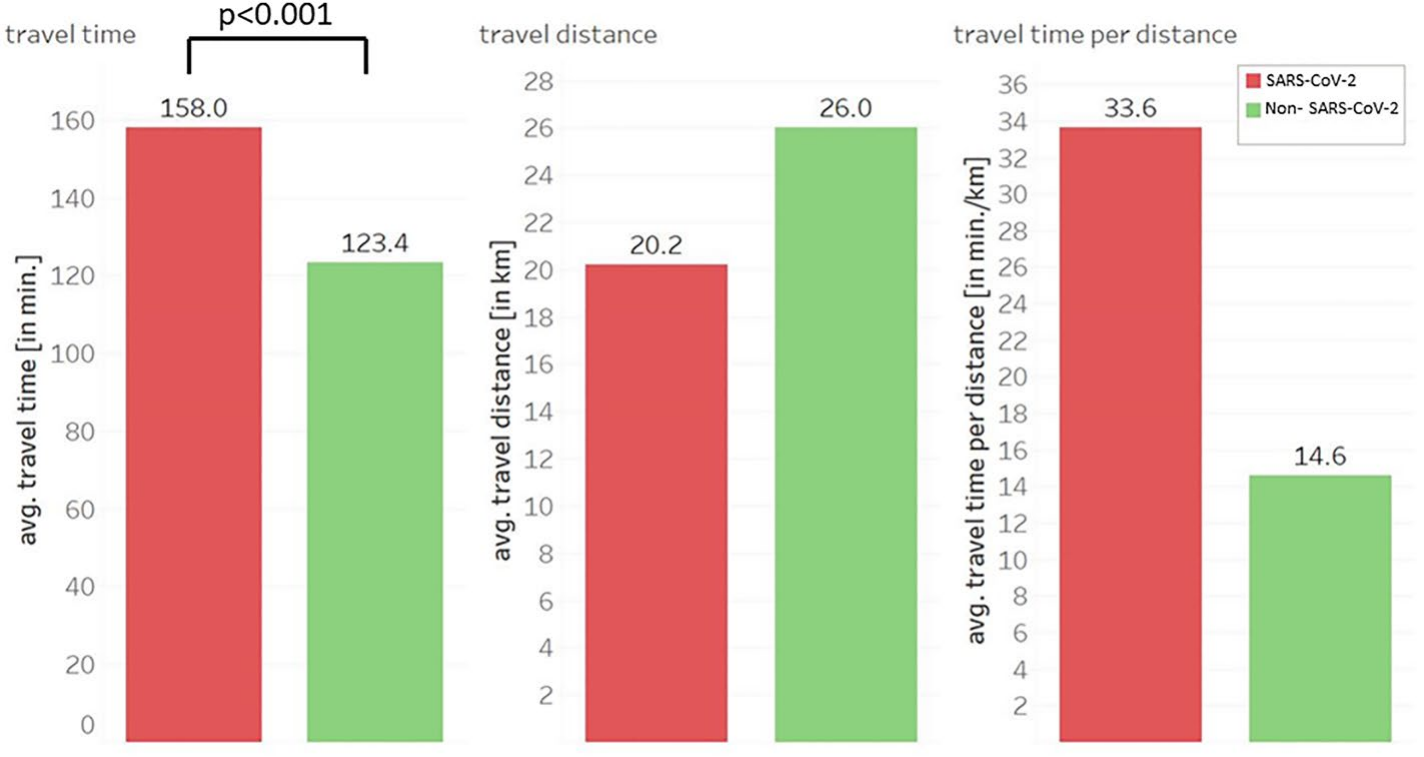
Comparison of travel time and distance to destination hospital between SARS-CoV-2 associated transports (red) and SARS-CoV-2 negative transports (green). avg… average.

A direct comparison of SARS-CoV-2 and non-SARS-CoV-2 associated transports shows that transports with infectious patients require more time despite shorter distances (158 min vs. 123 min) (Fig. 4). If the quotient of duration and distance is used, the two values diverge even further (33.6 min/km vs. 14.6 min/km). First, the Shapiro–Wilk test was applied to test whether a normal distribution can be assumed for the variable “travel time”. The test is significant (*p* value < 0.001), meaning that no normal distribution can be assumed. Thus, the Wilcoxon rank sum test is used to test whether the “travel time” of secondary transports is significantly different in the two groups (SARS-CoV-2 vs. non SARS-CoV-2). The Wilcoxon test and therefor the difference on “travel time” is significant (*p* value < 0.001).

A relevant factor in planning secondary transport is the age of transported patients (Table 4). The average age for SARS-CoV-2 patients is 5 years higher than the average age for non-infectious patients (69.6 and 64.8 years, respectively, *p* < 0.001). Analysing the age distribution in more detail, the proportion of patients in the age group “36–65 years” is almost identical for both groups (27.45% vs. 29.2%). On the other hand, there are more older patients over 65 years in the SARS-CoV-2 positive group (68.6% vs. 58.7%). In comparison, the proportion of younger patients (0–35 years) in the SARS-CoV-2- positive group is lower (3.1% vs. 10.8%).

**Table 4:** Age distribution of SARS-CoV-2 negative and SARS-CoV-2 positive patients (*p* < 0.001).

Age distribution (%)	0–35	36–65	66–95	> = 96
non-SARS-CoV-2	10.8	29.2	58.3	0.4
SARS-CoV-2	3.1	27.5	68.3	0.3

The Shapiro–Wilk test was applied to test whether a normal distribution can be assumed for the variable “age”. The test is significant (*p* value < 0.001) and the Wilcoxon rank sum test is performed. The test is also significant (*p* value < 0.001) for the arithmetic mean “69.6 years” (SARS-CoV-2) and “64.8 years” (non-SARS-CoV-2). The chi-square test is performed and it is also significant (*p* < 0.001). The age distribution is therefore significantly different in the two groups (Table 4).

Table 5 shows a summary of medical transport vehicles used for secondary transports of SARS-CoV-2 and non-SARS-CoV-2 patients. Non-infectious transports primarily use transport ambulances (KTW, 47.8%), emergency ambulances (RTW, 40.9%), medical helicopters (RTH, 6.2%) and intensive care transport vehicles (ITW, 1,2%). Infectious transfers primarily took place with emergency ambulances (RTW, 51.3%), followed by transport ambulances (KTW, 33.8%), intensive care transport vehicles (ITW, 6.5%) and medical helicopters (RTH, 4%). A direct comparison illustrates that emergency ambulances (RTW) are needed 10% more often for infectious patients than for non- infectious patients. In addition, more critical care transport vehicles (ITW) are used for COVID-19 patients than for non-COVID-19 patients (6.5% vs. 1.2%). The chi-squared test is performed showing that the distribution of the transport type is significantly different between the SARS-CoV-2 and the non- SARS-CoV-2 secondary transports (*p* < 0.001).

**Table 5:** Means of transport for non- SARS-CoV-2 and SARS-CoV-2 associated transports (*p* < 0.001).

Mean of transport (%)	KTW	RTW	RTH	NEF	ITW	ITH	RTW-infection
non-SARS-CoV-2	47.8	40.9	6.2	1.6	1.2	0.2	0.14
SARS-CoV-2	33.8	51.3	4	1.4	6.5	0	2.07

The distribution of secondary transports across weekdays shows that from Monday to Friday, SARS-CoV-2 and non-SARS-CoV-2 associated transports were about equally distributed (average 17.5% of transports every day) both starting with a lower number of transfers on Monday. Saturdays and Sundays account for only 6.65% % and 5.38% of non-infectious transports, respectively. In contrast, SARS-CoV-2 associated transports show a peak on Wednesday (20.31%) and are requested more frequently on Saturday (9.38%) and Sunday (6.98%) (Table 6). A detailed table showing the distribution of secondary transfers by weekdays and the corresponding means of transportation is provided in the Appendix (Supplement Table 1).

**Table 6:** Distribution of transport vehicles among days of the week for non-SARS-CoV-2 and SARS-CoV-2 associated transports (*p*=0.006).

	Monday	Tuesday	Wednesday	Thursday	Friday	Saturday	Sunday
non-SARS-CoV-2	15.5	16.8	18.2	17.8	17.7	6.6	5.4
SARS-CoV-2	15.1	17.2	20.3	15.4	14.8	9.4	7.0

A more detailed understanding of the dynamics of infections with SARS-CoV-2 would enable authorities to take precautions in the next wave to avoid overloading of the healthcare system. The analysis of secondary transports of SARS-CoV-2 patients depending on SARS-CoV-2 incidences and depending on hospital structure presents an opportunity to plan transport capacities in advance if the number of infections increases.

**Figure 5:**
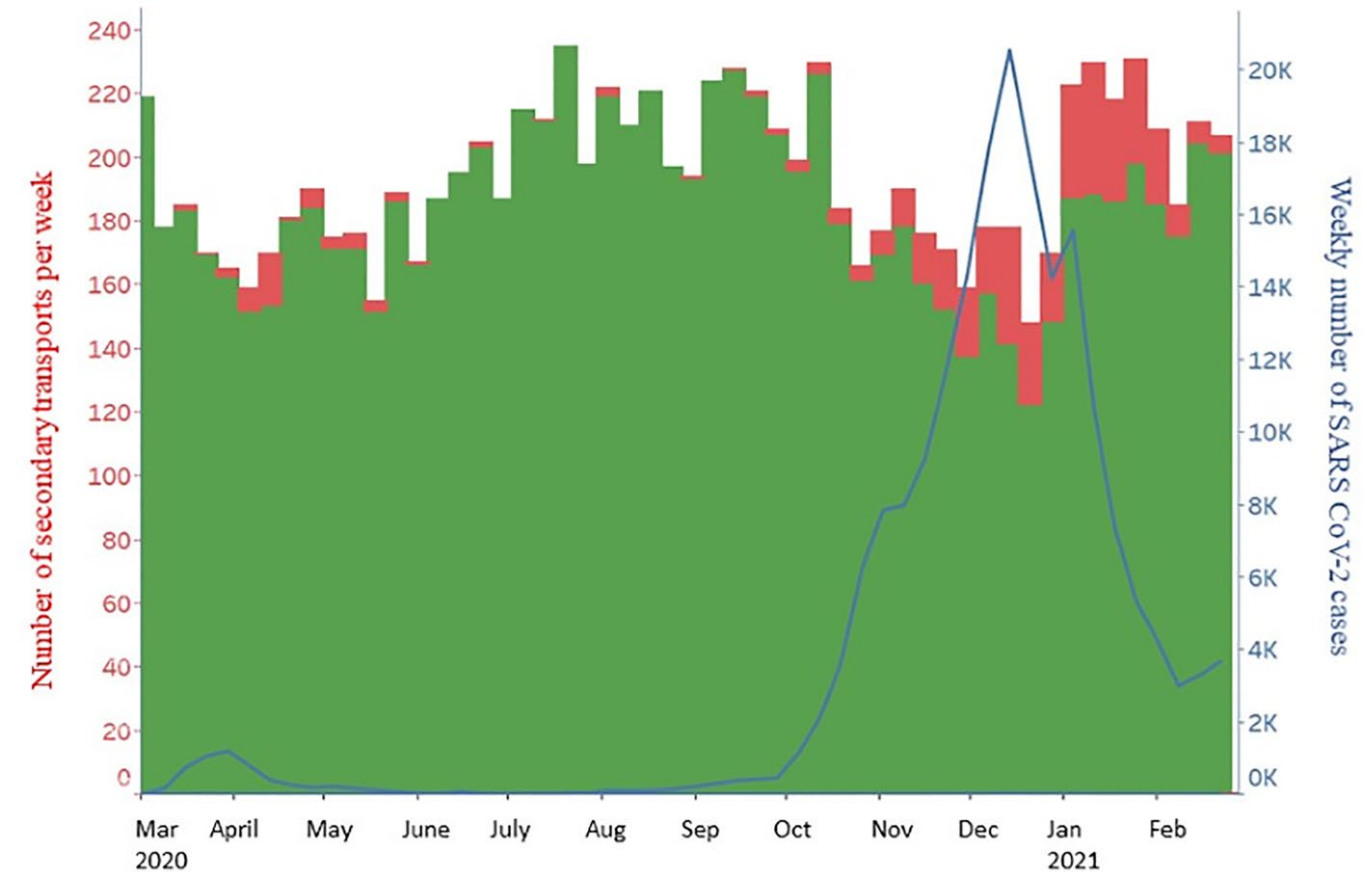
Number of all secondary transports per week (green and red columns) (left y-axis) in relation to weekly SARS-CoV-2 cases in Saxony (blue) (right y-axis) from March 2020 to February 2021.

As illustrated in Fig. 5, the number of all secondary transports declined during the waves of infection.

In contrast, SARS-CoV-2 associated transports increased parallel to the corresponding rise of weekly cases (Fig. 3, left side, red columns). Analysis of secondary transports by hospital type showed differences among hospitals.

**Figure 6:**
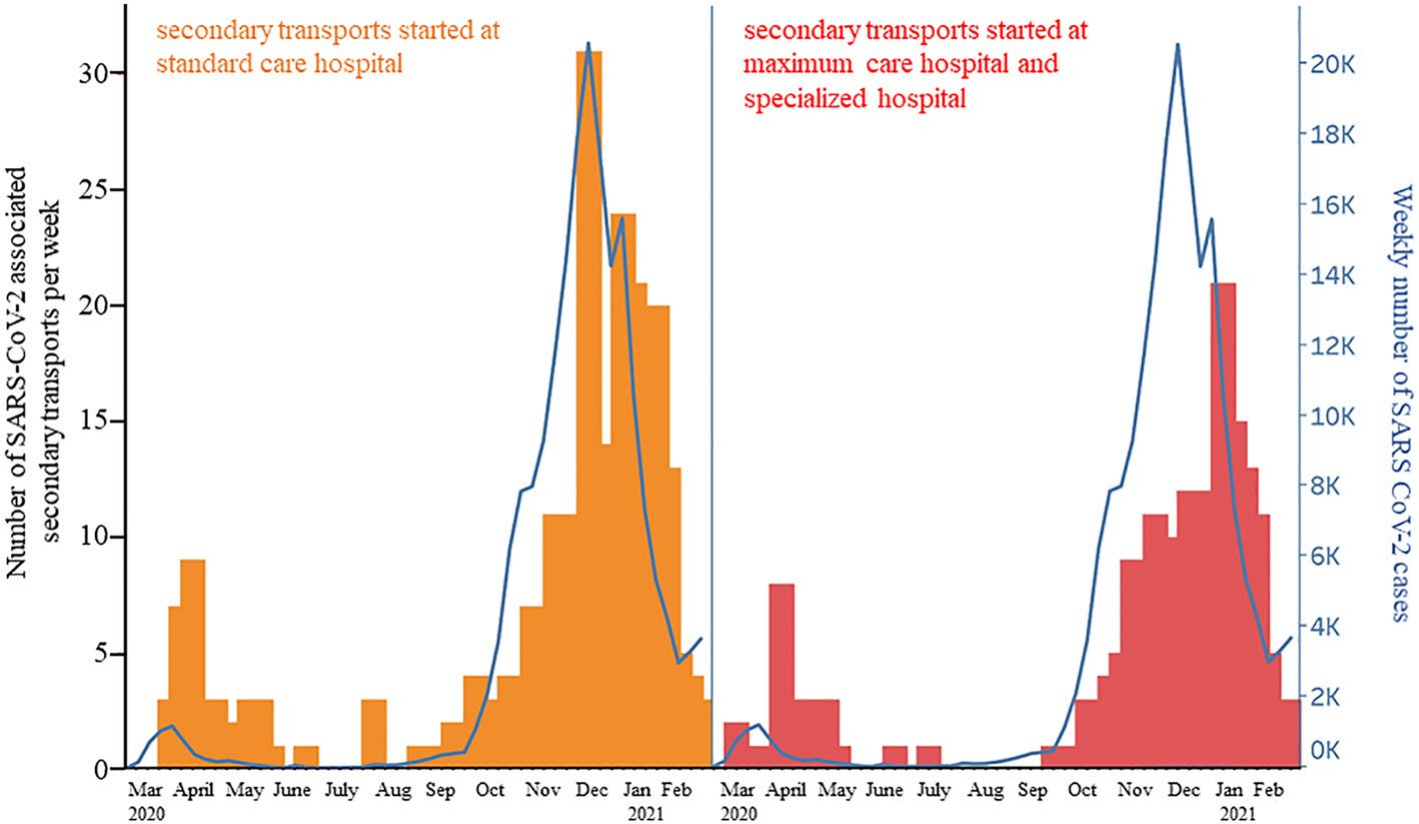
Number of SARS-CoV-2 associated secondary transports per week from standard care hospitals (orange columns, left y-axis) and maximum care and specialized hospitals (red colums, left y-axis) in relation to weekly SARS-CoV-2 cases in Saxony (blue) (right y-axis) from March 2020 to February 2021.

Figure 6 shows secondary transports of SARS-CoV-2 patients from standard care hospitals (left) and maximum care and specialized hospitals (right), respectively, in relation to weekly cases. Infectious patients left maximum care and specialized hospitals about 4 weeks after the highest incidences. In contrast, standard care hospitals transferred patients at the time of the highest SARS-CoV-2 incidences. The two peaks in weekly SARS-CoV-2 cases are accompanied by two peaks of increased secondary transport.

**Figure 7:**
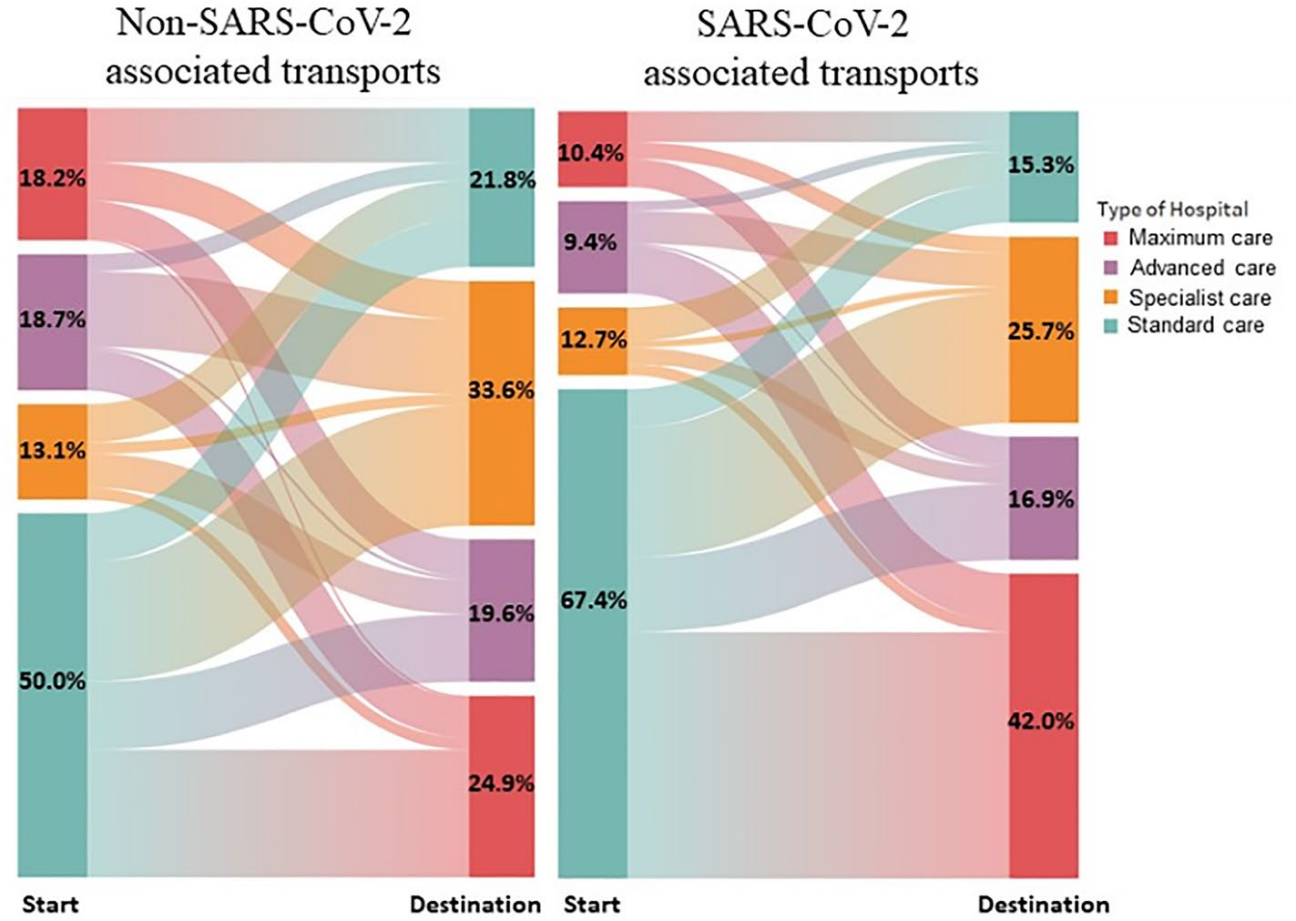
Overview of non-SARS-CoV-2 (left) and SARS-CoV-2 associated secondary transports (right) illustrating start and destination hospitals.

An overview of infectious and non-infectious secondary transports illustrates where different types of hospitals transferred their patients during the pandemic period March 2020 to February 2021 (Fig. 7). An increased transport volume of SARS-CoV-2 patients to maximum care hospitals is evident (42%, right panel, red, Destination). In addition, standard care hospitals discharged more SARS-CoV-2 patients (67.4%, right panel, green, Start) whereas the largest share of patients were transported to maximum care providers. Specialist hospitals and advanced care hospitals differed little in their transport patterns between infectious and non-infectious patients.

## Discussion

The Corona pandemic presented unprecedented challenges to healthcare systems worldwide. Highly developed industrialised countries like Germany had to reduce elective hospital care and nevertheless in part had to handle local patient surcharge. Germany has the highest number of intensive care beds in the OECD with almost 34 beds per 100,000 inhabitants^22^. Despite ideal conditions in terms of a highly effective health care system, the virus posed a considerable challenge to a country with little experience in widespread pandemic management.

In Germany, regional pandemic concepts were in the responsibility of the 16 federal states. Saxony was divided into three COVID-19 coordination clusters: Chemnitz, Dresden and Leipzig. Clusters coordinated secondary transports of SARS-CoV-2 patients first within a cluster and, if cluster resources were exhausted, then to one of the other clusters. Our study aim was the evaluation of secondary transport data of SARS-CoV-2 positive and negative patients in Saxony during the first and second Corona wave in 2020/2021. The state of Saxony had two characteristics that made evaluation of interhospital transfers feasible: (1) Secondary transports are centrally coordinated by a single control centre (IRCC), thus a complete collection of all transport date is available from one single location. (2) No other federal state had higher SARS-CoV-2 incidences, higher intensive care unit occupancies with COVID-19 patients or higher COVID-19 associated mortalities during the second wave in Germany. Our summary of the three entities (SARS-CoV-2 incidences, ICU occupancy, COVID-19 associated mortality) impressively shows the impact of COVID-19 in Saxony and thus the need for secondary transports in high incidence areas within a cluster.

Our data demonstrate that the total number of secondary transports was slightly reduced in the pandemic period. Because a SARS-CoV-2 associated transport takes an average of 1.28 times longer than a transport of non-infectious patients, overall transport capacities were comparable to the reference period (March 1st 2019 to February 28th, 2020). SARS-CoV-2 associated transports did not take place additionally, but the number of normal transports was reduced. This may have become necessary for purely capacity reasons, as there is only a limited number of transport vehicles available for patient transport. In-hospital measures such as postponing elective surgeries reduced planned caseloads and led to fewer non-infectious transports^9^. Otherwise, a compensation of additional SARS-CoV-2 associated secondary transports would not have been possible.

We could show that SARS-CoV-2 patients were older than non- infectious ones and that an infectious secondary transport took more time despite shorter distances. One reason for this could be that significantly sicker patients were transported who required more organisational time. As the emergency ambulance (RTW) was the first choice transport vehicle, it reinforces the assumption that patients were significantly sicker. Similar to our data, an analysis of intensive care transports (COVID-19 and non-COVID-19 patients) from Germany demonstrated that COVID-19 patients had increased disease severity and intensive care requirements^23^. More time was also needed during patient handover at the arrival hospital, as SARS-CoV-2- patients usually had a longer and complicated medical history. A third reason why SARS-CoV-2 associated transports took longer is the fact that transport vehicles must be extensively cleaned and disinfected after the journey.

Our transport data do not include the reason for transportation (quantitative or qualitative). The type of transport vehicle and the level of care of the two hospitals involved provide reasonable scenarios: secondary transports of SARS-CoV-2 patients with a standard ambulance from one regular care hospital to another were most likely necessary for capacity reasons. In contrast, secondary transports of SARS-CoV-2 positive patients with emergency ambulances or intensive care transport vehicles will most likely lead to a hospital with expanded medical expertise. A secondary transport of an intensive care patient represents an enormous medical risk and is not indicated thoughtlessly. Ligtenberg et al.^24^ demonstrated that one third of critically ill patients who had to be transferred to an intensive care unit due to a medical indication had critical events during transport that acutely endangered their lives. The WHO has recognized the high risk of secondary transports to both SARS-CoV-2 patients and accompanying medical personnel and has published guidelines to ensure safe transportation^25^.

The choice of transport is based on the severity of the patient's illness. For SARS-CoV-2 patients, emergency ambulances were the most frequently used means of transport, followed by transport ambulances and intensive care transport vehicles. Emergency ambulances are mainly used for patients who require extended medical monitoring or need to be transferred ventilated. This fact is especially important because SARS-CoV-2 transports did not only take place during weekdays, but more patients needed transfers during weekends.

The total number of secondary transports during the pandemic phase decreased a little compared to the reference period. When weekly SARS-CoV-2 numbers and SARS-CoV-2 associated secondary transports are directly correlated, secondary transfers show a parallel progression to case numbers. Selecting maximum care and specialized hospitals only, transfers peaked approximately 4 weeks after the highest case numbers. This wave of transfers from maximum care hospitals and the timing can be explained by the approximate length of stay of COVID-19 patients in hospital. By analysing hospital data from more than 10,000 SARS-CoV-2 positive patients from 920 hospitals, Karagiannidis et al. could show that ventilated patients (invasive and non-invasive) stay in hospital for approximately 25 days (almost 4 weeks)^26^. Acute convalescence is then likely to be complete and the phase of rehabilitation can begin. Standard care hospitals, in contrast, transferred their patients at the time of the highest SARS-CoV-2 incidences. The two peaks in weekly SARS-CoV-2 cases were accompanied by two peaks of increased secondary transport. A large proportion of transfers from standard care hospitals was to maximum care hospitals. Both medical and capacity reasons could have necessitated a transfer. Also, SARS-CoV-2 associated secondary transports to maximum care hospitals are increased compared to transfers of non-infectious patients. It is reasonable to assume that the care of critically ill COVID-19 patients, and thus an immense physical and psychological burden, was on the wards of maximum care hospitals.

One limitation is that the transport data do not include the reason for transportation. Patients with only SARS-CoV-2 colonization or patients whose infectious status had not yet been clarified at the time of transport, as well as patients severely ill with COVID-19, are marked as “SARS-CoV-2 transport”. Qualitative or quantitative reasons for interhospital transfers can only be derived indirectly.

The initial dataset consisted of 37,815 secondary transports from March 2019 to February 2021. Due to our exclusion criteria, the final data set was reduced to 25,175 interhospital transfers. Thus, we have excluded about one third of secondary transports from our analysis, such as transfers to other federal states or abroad. These excluded patients have SARS-CoV-2/COVID-19 but are eligible for prolonged transport as they are (presumably) cardiopulmonarily stable (analogous to the cloverleaf transfer concept^27^). Assuming that respiratory and cardiac unstable patients were more likely to be transferred within Saxony, this patient clientele might be disproportionately represented in our analysis.

In our analysis, we excluded all transfers in case a hospital or hospital-group has several locations. It cannot be ruled out that reasons other than those mentioned above took priority in these transfers. Also, these transport cases may not have been regular secondary transfers from one hospital to another, but from one building to another building within a hospital group. We wanted to rule out this bias and excluded transfers of hospitals or hospital-groups with several locations.

Another limitation is that data from Saxony may not be applicable in other federal states or abroad as the organisation of secondary transports is handled differently. In Germany, in-patients are free to choose their hospital for primary health care. The nearest hospital is usually the initial contact point- regardless of the hospital type. In rural areas, standard care hospitals are predominant, providing basic internal medicine and surgical care and offer a limited number of intensive care beds. On the other hand, in-patients in large cities have direct access to advanced care or maximum care hospitals without the need for interhospital transports in case of therapy escalation or capacity problems. Hospital dispatch centres (such as the IRCC in Saxony) are responsible for the organisation of secondary transports and the efficient distribution of patients. In other countries (e.g., in Scandinavia), primary care is provided in the periphery and patients are regularly transferred to maximum care hospitals. The SARS-CoV-2 pandemic caused congestion in health care systems across Europe, and alternative transportation options were developed to relieve regional hospitals (i.e. ambulance bus in the Netherlands^28^ or transport by civilian helicopter emergency medical service (HEMS, UK)^29^.

The evaluation of data on secondary transport in Saxony during the first and second SARS-CoV-2 wave highlights the critical infrastructure “transport system” as an essential component for achieving the optimal therapy for patients. Coordination of secondary transport should be adjusted (in future pandemics) to the number and severity of patients and should not be a limiting component. One future goal would be to develop a more efficient and resource-saving concept in order to be able to plan the number of transport capacities in advance as soon as the number of infections increases.

## Conclusion

The extent of a corona wave is determined by the predominant viral variant (at that time) and the associated disease severity. Our data clearly demonstrate that—depending on disease severity and SARS-CoV-2 incidence—there is a direct correlation to the need for secondary transport. Our results could facilitate planning of transport capacity in future waves to anticipate and adjust both the type and number of transport vehicles. Significantly more and specific transport vehicles as well as medical staff should be planned particularly over weekends during a wave of infection. In addition, intensive care transport vehicles can be concentrated or proactively requested in regions where there is a disproportionate increase in infection rates.

The pattern of interhospital transports shows initial transfers from the periphery to maximum care hospitals and, with a delay of 4 weeks, transfers away from maximum care providers.

Our findings could enable more predictive planning of transport capacities. Depending on viral variants and incidences, the Integrated Rescue Control Centre may be “on alert” when SARS-CoV-2 numbers increase and proactively keep sufficient transport resources on standby.

## Supplementary Information

Supplementary Table 1.

## Data Availability

Data retrieval is possible by contacting Yacin Keller (ykeller@dresden.de) or Ulrich Pankotsch (UPankotsch@Dresden.de) from the Integrated Rescue Control Center (“Integrierte Rettungsleitstelle”, IRLS) in Dresden. Data can be retrieved by specifying the reason for data processing, after written agreement and as long as German data protection law can be complied with.

## Abbreviations

Avg: Average
ARDS: Acute respiratory distress syndrome
COVID-19: COronaVIrus disease 2019
CVC: Central venous catheter
DIVI: Deutsche interdisziplinäre vereinigung für intensiv- und notfallmedizin
ECMO: ExtraCorporeal membrane oxygenation
HEMS: Helicopter emergency medical service
ICD-10: International statistical classification of diseases and related health problems
ICU: Intensive care unit
ITW: Intensivtransportwagen (intensive care transport vehicle)
IRCC: Integrated rescue control centre
KTW: Krankentransportwagen (transport ambulance)
MCI: Mass casualty incident
NEF: Notarzteinsatzfahrzeug (emergency doctor’s vehicle)
NUM: Netzwerk Universitätsmedizin, Network University Medicine
NUTS: Nomenclature des unités territoriales statistiques
OECD: Organisation for economic co-operation and development
RKI: Robert Koch Institute
RTH: Rettungshubschrauber (rescue helicopter)
RTW: Rettungswagen (emergency ambulance)
SARS-CoV-2: Severe acute respiratory syndrome CoronaVirus type 2
WHO: World Health Organisation
