# Supplementary material for: Burden of hospital admissions and resulting patient interhospital transports during the 2020/2021 SARS-CoV-2 pandemic in Saxony, Germany
Source: Sci Rep. 2023 May 24;13:8407. doi: 10.1038/s41598-023-35406-y (PMC10206557; doi:10.1038/s41598-023-35406-y)
Supplement: Supplementary file 1 — Supplementary Table 1. [file 41598_2023_35406_MOESM1_ESM.pptx]

## Slide 1
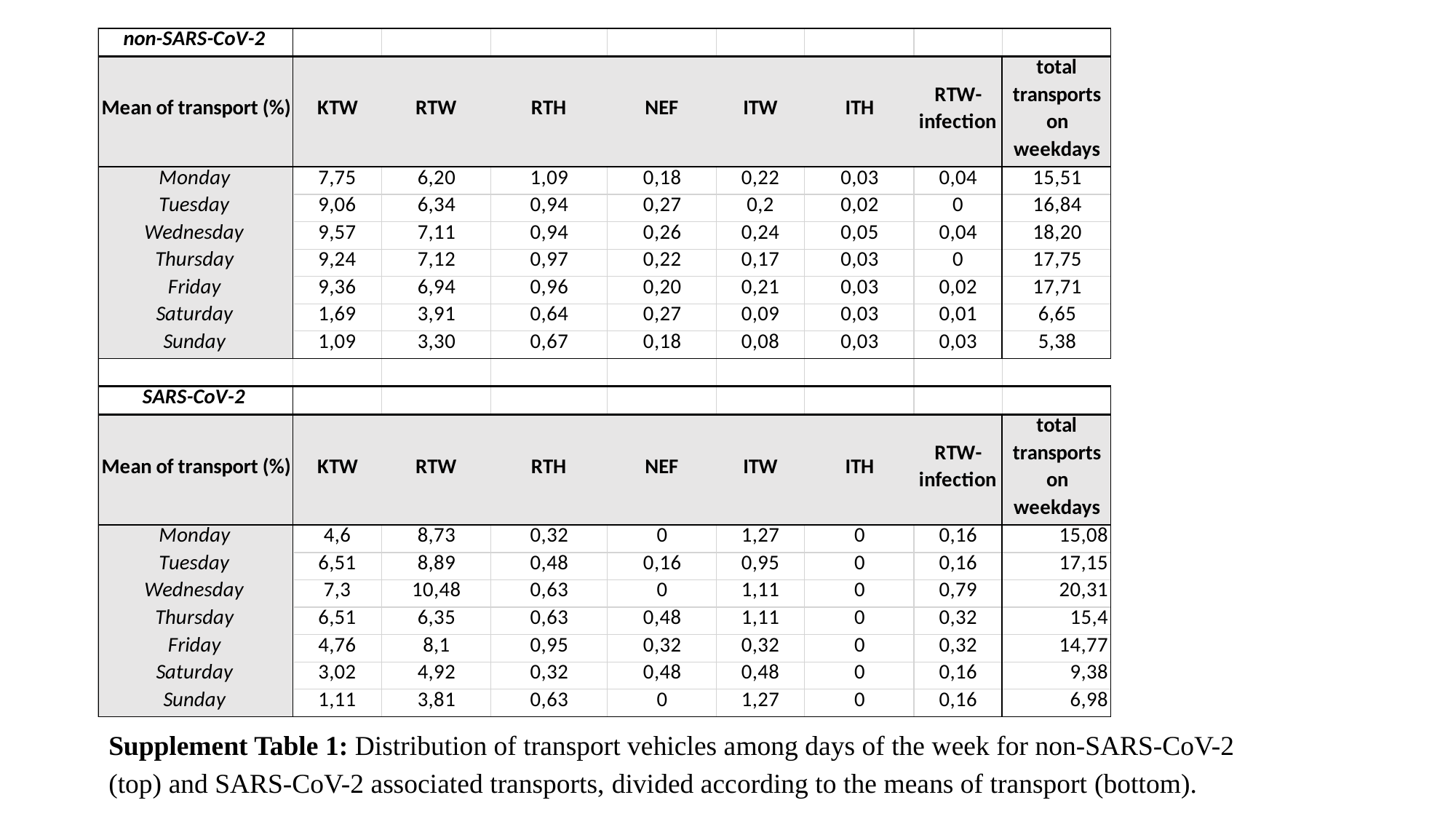

Supplement Table 1: Distribution of transport vehicles among days of the week for non-SARS-CoV-2 (top) and SARS-CoV-2 associated transports, divided according to the means of transport (bottom).
